# Non-Electrophilic Activation of NRF2 in Neurological Disorders: Therapeutic Promise of Non-Pharmacological Strategies

**DOI:** 10.3390/antiox14091047

**Published:** 2025-08-25

**Authors:** Chunyan Li, Keren Powell, Luca Giliberto, Christopher LeDoux, Cristina d’Abramo, Daniel Sciubba, Yousef Al Abed

**Affiliations:** 1 Translational Brain Research Laboratory, Feinstein Institutes for Medical Research, Manhasset, NY 11030, USA; 2 Institute of Bioelectronic Medicine, Feinstein Institutes for Medical Research, Manhasset, NY 11030, USA; 3 Department of Neurosurgery, Zucker School of Medicine at Hofstra/Northwell, Hempstead, NY 11549, USA; 4 The Litwin-Zucker Center for Alzheimer’s Disease & Memory Disorders, Institute of Molecular Medicine, Feinstein Institutes for Medical Research, Manhasset, NY 11030, USA; 5 Institute for Neurology and Neurosurgery, Northwell Health, Manhasset, NY 11030, USA; 6 Department of Biology, Hofstra University, Hempstead, NY 11549, USA

**Keywords:** NRF2, electrophilic, non-electrophilic, neurological disorders, non-pharmacological intervention, neuromodulation

## Abstract

Nuclear factor erythroid 2-related factor 2 (NRF2) serves as a master transcriptional regulator of cellular antioxidant responses through orchestration of cytoprotective gene expression, establishing its significance as a therapeutic target in cerebral pathophysiology. Classical electrophilic NRF2 activators, despite potent activation potential, exhibit paradoxically reduced therapeutic efficacy relative to single antioxidants, attributable to concurrent oxidative stress generation, glutathione depletion, mitochondrial impairment, and systemic toxicity. Although emerging non-electrophilic pharmacological activators offer therapeutic potential, their utility remains limited by bioavailability and suboptimal potency, underscoring the imperative for innovative therapeutic strategies to harness this cytoprotective pathway. Non-pharmacological interventions, including neuromodulation, physical exercise, and lifestyle modifications, activate NRF2 through non-canonical, non-electrophilic pathways involving protein–protein interaction inhibition, KEAP1 degradation, post-translational and transcriptional modulation, and protein stabilization, though mechanistic characterization remains incomplete. Such interventions utilize multi-mechanistic approaches that synergistically integrate multiple non-electrophilic NRF2 pathways or judiciously combine electrophilic and non-electrophilic mechanisms while mitigating electrophile-induced toxicity. This strategy confers neuroprotective effects without the contraindications characteristic of classical electrophilic activators. This review comprehensively examines the mechanistic underpinnings of non-pharmacological NRF2 modulation, highlighting non-electrophilic activation pathways that bypass the limitations inherent to electrophilic activators. The evidence presented herein positions non-pharmacological interventions as viable therapeutic approaches for achieving non-electrophilic NRF2 activation in the treatment of cerebrovascular and neurodegenerative pathologies.

## 1. Introduction

**Figure 1:**
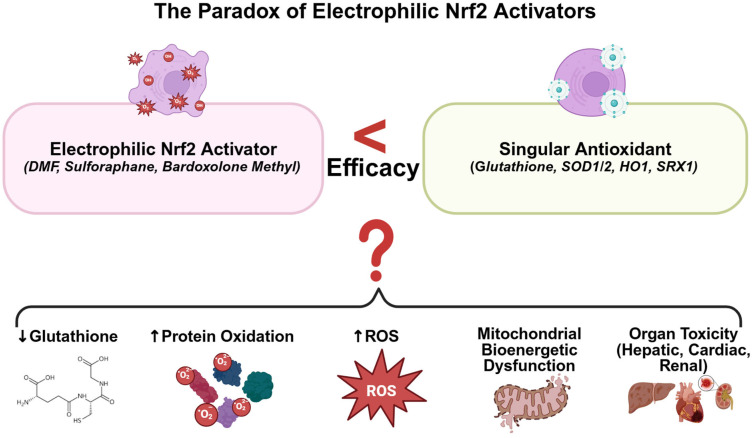
Paradoxical consequences of electrophilic NRF2 activation. Although NRF2 coordinates the transcription of hundreds of antioxidant and cytoprotective genes, single antioxidant agents can, paradoxically, outperform classical electrophilic NRF2 inducers in therapeutic settings. This diminished efficacy arises from liabilities inherent to electrophilic compounds, including (1) depletion of intracellular glutathione, (2) exacerbation of protein oxidation, (3) amplification of reactive oxygen species, (4) disruption of mitochondrial bioenergetics, and (5) off-target organ toxicity—most notably hepatic, cardiac, and renal injury. These contraindications highlight the imperative to investigate non-electrophilic NRF2 activators. (Created with BioRender.com).

Nuclear factor erythroid 2-related factor 2 (NRF2) functions as a master transcriptional regulator of cellular antioxidant defense, governing the expression of hundreds of cytoprotective and detoxifying genes [1,2,3,4]. While NRF2 activation presents an attractive therapeutic strategy capable of coordinating multi-antioxidant responses, emerging evidence challenges this paradigm. Despite documented beneficial effects, accumulating data demonstrate that classical NRF2 activators exhibit suboptimal efficacy against oxidative damage when compared to single antioxidant interventions [5,6,7] (Figure 1), necessitating critical re-evaluation of current approaches. Under normal physiological conditions, pharmacological NRF2 activators have paradoxically been shown to deplete glutathione [6], compromise mitochondrial function [8], and elevate reactive oxygen species (ROS) levels despite potent NRF2 pathway activation. In chronic pathological settings, where these disturbances intersect with pre-existing cellular damage, their effects become highly variable, ranging from diminished therapeutic benefit to an aggravation of tissue injury and acceleration of disease progression [6,7,9,10,11,12]. Notably, dimethyl fumarate (DMF) accelerates glutathione depletion in multiple sclerosis [9], further driving the observed downregulation of glutathione in multiple sclerosis [10], and potentially causing hepatic injury even with acute application. This paradox intensifies in severe, acute, neurological injury: DMF and sulforaphane exacerbate protein oxidation and infarct expansion [6,13,14,15] relative to glutathione [7] or superoxide dismutase (SOD) mimetics [11,12], occasionally yielding inferior outcomes versus untreated controls in stroke models [15]. Traumatic brain injury studies corroborate these findings, with DMF augmenting lipid peroxidation and compromising neuroprotection [16,17,18]. These observations suggest that, under specific conditions, classical NRF2 activators may fail to achieve anticipated therapeutic benefits and potentially compromise neural integrity. Consequently, mechanistic dissection of this paradox and identification of alternative NRF2 activation strategies constitute essential steps toward realizing the full therapeutic potential of this critical cytoprotective pathway.

**Figure 2:**
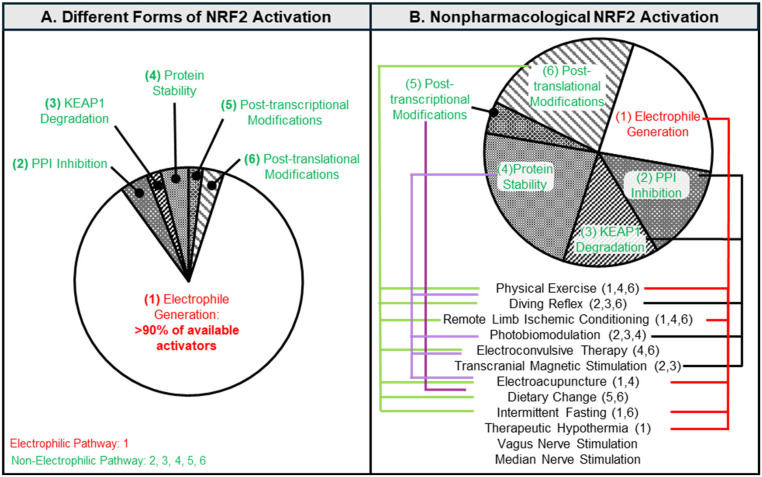
Mechanistic spectrum of NRF2 activation. (**A**) NRF2 can be activated through six principal routes: (1) non-specific electrophile/ROS generation, (2) disruption of the NRF2–KEAP1 protein–protein interface, (3) autophagy-driven KEAP1 degradation, (4) direct stabilization of NRF2, (5) post-transcriptional regulation, and (6) post-translational modification. Despite the fact that only pathway 1 is electrophilic, >90% of currently available small-molecule activators exploit electrophilic chemistry. (**B**) Non-pharmacological NRF2 inducers display a markedly different mechanistic distribution: they are enriched for non-electrophilic pathways and often engage multiple mechanisms concurrently. Of the twelve non-pharmacological interventions assessed in this review—excluding the two whose mechanisms have yet to be elucidated—only therapeutic hypothermia operates purely through an electrophilic mechanism. The four other interventions that exhibit some degree of electrophilicity do so in conjunction with additional, non-electrophilic pathways, highlighting their mechanistic divergence from conventional electrophile-based activators.

Current NRF2 activators predominantly employ electrophilic mechanisms (Figure 2), requiring ROS generation to dissociate NRF2 from the NRF2:Kelch-like ECH-associated protein 1 (KEAP1) complex [19]. This ROS-mediated activation depletes glutathione reserves, increases protein oxidation and lipid peroxidation, and disrupts mitochondrial bioenergetics [1,2,3,4,5,6,7,20,21,22,23,24]. While metabolically intact cells may tolerate this oxidative burden, it poses significant risks to cells experiencing oxidative stress, particularly during conditions demanding rapid, pronounced NRF2 activation and in highly vulnerable tissues such as the brain. The brain exhibits exceptional metabolic demands, consuming 20% of total oxygen despite comprising only ~2% of body mass [5]. This disproportionate oxygen utilization for glucose-dependent ATP synthesis inherently generates substantial ROS. Combined with high lipid content, limited antioxidant capacity, and dense neuronal populations, these characteristics render the brain uniquely susceptible to oxidative damage [25]. Neurons demonstrate particular vulnerability due to their dependence on mitochondrial metabolism and elevated mitochondrial ROS production [20,21,22,23,24,26,27,28,29,30], making them highly sensitive to electrophile-induced bioenergetic dysfunction. Glutathione depletion precipitates neurodegeneration [31,32], contributing to neurodegenerative diseases, chronic neurological dysfunction, and psychological disorders while increasing susceptibility to acute traumatic injury [33,34]. The brain’s intrinsic oxidative vulnerability may therefore explain the compromised efficacy of electrophilic NRF2 activators, as their ROS-dependent mechanism [19,35,36] exacerbates existing oxidative stress. Consequently, developing non-electrophilic NRF2 activation strategies represents an essential requirement for safely exploiting NRF2’s neuroprotective potential.

Recent investigations have increasingly focused on non-electrophilic NRF2 activation strategies. Unlike conventional approaches that induce NRF2 through non-specific ROS generation, non-electrophilic activators employ targeted mechanisms including protein–protein interaction (PPI) disruption, KEAP1 degradation, protein stabilization to enhance NRF2 bioavailability, and post-translational/post-transcriptional modifications to augment NRF2 function [37,38,39] (Figure 2). Despite theoretical advantages, these approaches exhibit notable limitations. Compounds promoting KEAP1 proteasomal degradation demonstrate efficacy comparable to electrophilic activators but remain scarce. While PPI inhibitors show therapeutic potential in diabetic wound healing and lipopolysaccharide-induced inflammation [39,40], they typically exhibit reduced potency relative to electrophilic compounds, similar to the efficacy observed with modulators targeting post-translational/transcriptional processes [38]. This diminished efficacy likely reflects the critical role of NRF2 bioavailability as a rate-limiting factor, which selective PPI inhibitors and post-translational/transcriptional modulators fail to enhance sufficiently compared to electrophilic agents. Consequently, identifying novel non-electrophilic activators that either promote KEAP1 degradation or integrate multiple mechanisms to simultaneously enhance NRF2 bioavailability and activity remains imperative. Non-pharmacological interventions represent a promising research direction, having demonstrated clinical efficacy in oxidative-stress-mediated cerebral pathologies [41] and NRF2 activation capacity [42,43,44,45,46,47]. Substantial evidence indicates these interventions operate through non-electrophilic pathways, including modulation of glycogen synthase kinase 3β (GSK3β), AMP-activated protein kinase (AMPK), and p38 mitogen-activated protein kinase (p38 MAPK), among others [42,43,44]. Their multi-mechanistic action may circumvent efficacy limitations inherent to single-target non-electrophilic approaches. Despite compelling evidence supporting their therapeutic utility and non-electrophilic mechanisms, however, non-pharmacological NRF2 activators remain inadequately investigated for neurological applications. 

This review comprehensively examines the therapeutic potential of non-pharmacological NRF2 activation in neurological disorders, with particular emphasis on non-electrophilic mechanisms and multi-target approaches. Our investigation addresses three critical questions: (1) whether non-pharmacological interventions operate through non-electrophilic pathways; (2) whether they exhibit limitations similar to electrophilic activators; and (3) whether they overcome limitations inherent to current non-electrophilic agents. We systematically evaluate effects and mechanisms across distinct pathophysiological contexts—physiological conditions, chronic pathology, and acute injury. The review investigates whether non-pharmacological interventions generate distinct neurological outcomes compared to pharmacological NRF2 activators, whether their activation mechanisms and downstream signaling are optimized for the brain’s unique metabolic milieu, and whether they represent substantive mechanistic innovations beyond current strategies. Observations from peripheral tissues are analyzed as potential indicators of central nervous system efficacy. We further examine how multi-mechanistic NRF2 activation by non-pharmacological interventions confers therapeutic advantages for clinical translation. Through critical evaluation of non-electrophilic, non-pharmacological approaches, this review identifies promising directions for mechanistic research and therapeutic development in NRF2-targeted neurological interventions.

## 2. Mechanistic Characterization of Non-Pharmacological NRF2 Activation: Strategic Implementation of Non-Electrophilic Pathways

Non-pharmacological NRF2 activators operate through non-electrophilic mechanisms, either partially or completely, and demonstrate therapeutic efficacy in conditions where conventional electrophilic activators exhibit suboptimal therapeutic indices [42,43,44,45,46,47]. These interventions show substantial potential to circumvent the limitations of electrophilic NRF2 activators while preserving the beneficial effects of NRF2 activation in disease states. To elucidate this paradigm, we first examine the current landscape of classical electrophilic pharmacological interventions, followed by a comprehensive analysis of non-pharmacological approaches. Particular emphasis is placed on their non-electrophilic mechanisms and capacity to address inherent limitations of conventional Nrf2 activators. Non-pharmacological interventions are systematically categorized according to the oxidative stress context: (i) absence of oxidative stress, (ii) gradually developing oxidative stress, and (iii) acutely developing oxidative stress from immediate causative factors. This organizational framework directly addresses critical concerns regarding the application of classical electrophilic NRF2 activators under conditions of elevated cellular stress or during chronic therapeutic regimens.

### 2.1. Electrophilic NRF2 Activation: Mechanistic Paradoxes and Therapeutic Limitations

Electrophilic NRF2 activators demonstrate substantial potency, inducing 1.5- to 3-fold increases in NRF2 activity [1,2,3,4,6,19], conferring theoretical therapeutic efficacy. However, the reactive promiscuity inherent to non-selective electrophiles constrains their clinical utility in neurological disorders (Figure 1). In cerebral pathologies, electrophilic activators paradoxically elevate oxidative stress markers/mediators concurrent with NRF2 induction and antioxidant upregulation [6], compromising therapeutic indices and potentially inducing organ toxicity. Mechanistically, oxidative modification of cysteine 151 dissociates NRF2 from the NRF2:KEAP1 complex, preventing ubiquitination and proteasomal degradation. This process generates elevated electrophile concentrations that may deplete antioxidant reserves before therapeutic benefits manifest in neural tissue [37,38]. Indeed, glutathione depletion represents the primary mechanism underlying DMF’s efficacy in psoriasis [48,49]. In neural tissue, this depletion proves particularly detrimental through downstream mitochondrial dysfunction [9]. Glutathione depletion restricts antioxidative capacity, elevating mitochondrial ROS and enhancing mitochondrial stress responses, ultimately compromising organellar function. This particularly affects neurons given their mitochondrial dependence and inherent oxidative vulnerability [50]. Consequently, under elevated oxidative stress conditions, electrophilic NRF2 activators yield negligible benefits, null effects, or paradoxically exacerbate pathological presentations [6,7,13,14,15]. Furthermore, chronic administration of electrophilic activators may induce organ toxicity, even under conditions of minimal baseline oxidative burden [38,51,52,53,54].

### 2.2. Non-Pharmacological NRF2 Activation Under Physiological Conditions: Mechanistic Insights in the Absence of Oxidative Stress

Unlike electrophilic NRF2 activators associated with organ toxicity, non-pharmacological approaches demonstrate favorable safety profiles without organ damage or mitochondrial bioenergetic disruption. Electrophilic NRF2 activators present limitations even under physiological conditions despite their potency. Notably, electrophilic compounds can substantially reduce glutathione levels within 60 min of administration [55,56,57]. Sulforaphane administration, while effectively inducing NRF2 [58], produces a 6-fold increase in brain nitrotyrosine in healthy animals [6]. However, absent pre-existing pathology, these paradoxical effects produce minimal adverse outcomes, as evidenced by widespread consumption of NRF2-activating polyphenolic compounds as dietary supplements [59,60,61,62].

**Figure 3:**
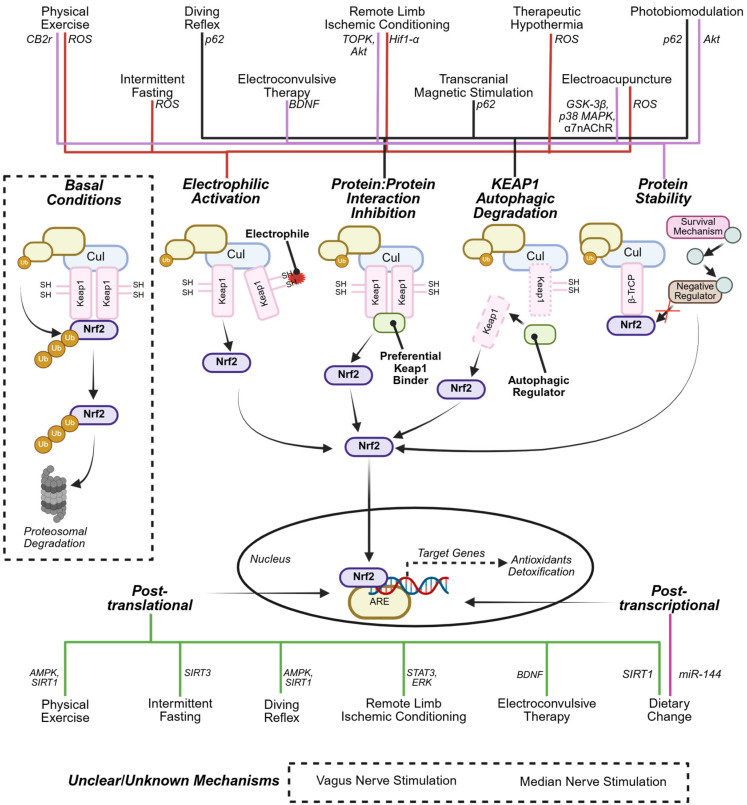
Non-pharmacological modalities predominantly engage multiple NRF2-activating pathways. NRF2 can be induced via: (i) non-specific electrophile/ROS generation, (ii) disruption of the NRF2–KEAP1 protein–protein interaction, (iii) autophagy-mediated KEAP1 degradation, (iv) direct modulation of NRF2 protein stability, and (v) post-transcriptional/post-translational modifications. Except for a single intervention, therapeutic hypothermia, every non-pharmacological strategy with defined mechanisms employs more than one of these routes, most frequently pairing post-translational modification with either protein-stability regulation or limited electrophile production. This combinatorial activation elevates both NRF2 abundance and transcriptional competence while minimizing the liabilities of purely electrophilic agents and circumventing the efficacy limitations reported for current single-mechanism, non-electrophilic compounds. (Created with BioRender.com) (abbreviations: α7nACHr: alpha7 nicotinic acetylcholine receptor; Akt: protein kinase B; AMPK: AMP-activated protein kinase; BDNF: brain-derived neurotrophic factor; CB2r: Cannabinoid receptor 2; ERK: extracellular signal-regulated kinase; GSK-3β: glycogen synthase kinase 3 beta; miR-144: microRNA-144; p38 MAPK: p38 mitogen-activated protein kinase; p62; ROS: reactive oxygen species; SIRT1: sirtuin 1; SIRT3: sirtuin 3; STAT3: signal transducer and activator of transcription 3; TOPK: T-LAK cell-originated protein kinase).

Multiple non-pharmacological interventions demonstrate NRF2 activation in physiological contexts, with many exhibiting partially or completely non-electrophilic mechanisms (Table 1, Figure 3). These include physical exercise, transcranial magnetic stimulation (TMS), therapeutic hypothermia (TH), diving reflex (DR), remote ischemic conditioning (RIPreC), photobiomodulation (PBM), intermittent fasting (IF), and dietary modifications. These interventions consistently induce significant NRF2 pathway activation across various metrics. Short-term physical exercise (≤2 days) elevates NRF2 levels and NRF2/antioxidant response element (ARE) binding activity, significantly increasing superoxide dismutase 1 and 2 (SOD1, SOD2), catalase, and heme oxygenase-1 (HO-1) expression [63,64,65]. The response demonstrates duration-dependence, with 6-h treadmill sessions producing more pronounced effects than 1-h sessions [64]. Similarly, DR exhibits time-dependent NRF2 activation, with enhanced NRF2 phosphorylation after 28-day treatment compared to single sessions, though acute treatment produces greater antioxidant gene upregulation [66].

Analysis of 19 documented non-pharmacological interventions under physiological conditions demonstrates consistent NRF2 activation. However, mechanistic understanding in healthy contexts remains limited. TMS and PBM activate p62, which competitively binds KEAP1 to release NRF2 without electrophile generation, while promoting autophagic KEAP1 degradation [44,67]. PBM upregulates NRF2 and LC3 expression in adipose-tissue-derived stem cells (ADSCs) [67] and reduces KEAP1 in human periodontal ligament stem cells [68]. DR employs similar non-electrophilic mechanisms through p62, AMPK, sirtuin 1 (SIRT1), and phosphoinositide 3-kinase (PI3K) induction, resulting in quinquepartite modulation: KEAP1 degradation, NRF2 protein–protein interaction inhibition, enhanced NRF2 stability, and increased DNA binding and transcription [66]. In contrast to TMS, PBM, and DR, physical exercise and IF exhibit dual activation mechanisms, incorporating both electrophilic and non-electrophilic pathways through ROS generation [63,64,69]. However, these interventions do not induce glutathione depletion, potentially due to concurrent non-electrophilic mechanisms including TLR4 and ZEB1 upregulation with ZEB2 downregulation [70,71].

### 2.3. Non-Pharmacological NRF2 Activation Under Conditions of Progressive Oxidative Stress

The presence of progressive oxidative stress significantly affects the efficacy and safety profile of electrophilic NRF2 activators. A critical translational limitation of electrophilic NRF2 activators involves cumulative cytotoxicity during chronic administration, manifesting as hepatotoxicity, nephrotoxicity, and cardiotoxicity [72,73,74,75]. DMF treatment in multiple sclerosis exemplifies this phenomenon, where patients exhibit elevated serum aminotransferases and bilirubin, progressing to severe acute hepatitis with hepatocellular bridging necrosis, even without pre-existing hepatic pathology [72,74,75,76]. Despite immediate withdrawal of DMF upon detection of elevated hepatic enzymes, liver injury may nonetheless progress. Notably, the incidence of DMF-associated hepatotoxicity or related adverse events increases markedly after at least three months of exposure, whereas patients with shorter treatment durations exhibit a substantially lower risk [73]. By contrast, omaveloxolone therapy in Friedreich’s ataxia has, to date, been accompanied only by mild aminotransferase elevations that are readily managed by dose modification [77]. Paradoxically, DMF administration in psoriasis patients induces only minor hepatic enzyme increases [78], whereas in multiple sclerosis populations it is associated with a higher rate of acute hepatotoxicity [76]. This differential toxicity correlates with varying oxidative stress burdens: despite both existing as autoimmune diseases, multiple sclerosis increases advanced oxidation protein products by ~24%, reduces thiol groups by ~49%, and diminishes ferric reducing ability by ~31% [75], whereas psoriasis demonstrates inconsistent advanced oxidation protein product modulation and only ~12% thiol group reduction [79,80,81]. Omaveloxolone’s favorable safety profile may reflect the relatively low oxidative-stress milieu in Friedreich’s ataxia. Although oxidative stress contributes to disease pathology, this disorder is not characterized by excessive reactive oxygen species; rather, frataxin-deficient cells display increased sensitivity to physiological oxidant levels [82]. By contrast, Phase III trials of bardoxolone methyl in chronic kidney disease were terminated due to serious cardiovascular adverse events [72]. The oxidative-stress-associated sarcopenia characteristic of chronic kidney disease [83] potentially increases cardiac vulnerability to cytotoxic insults. Collectively, these observations support a dose–response relationship between baseline oxidative stress levels and the toxicity of electrophilic activators, implying the existence of a threshold of oxidant burden beyond which these agents become contraindicated. In contrast, non-pharmacological NRF2 activators exhibit a favorable safety profile and preliminary signs of efficacy in pre-clinical models of chronic disorders characterized by progressive oxidative stress (Table 2), with particularly promising results in the brain.

Non-pharmacological interventions effectively induce NRF2 activation across diverse chronic pathologies, including non-alcoholic fatty liver disease, parkinsonism, Huntington’s disease, vascular dementia, Alzheimer’s disease (AD), post-traumatic stress disorder (PTSD), and chronic unpredictable mild stress (CUMS) (Table 2, Figure 3). Electroacupuncture activates NRF2 in complex regional pain syndrome type-1, colitis, sepsis, and diabetic encephalopathy [84,85,86,87]. Physical exercise demonstrates therapeutic benefit in parkinsonism, high-fat diet (HFD)-induced non-alcoholic fatty liver disease, and senescence, upregulating NRF2 and SOD1 expression while reducing lipid peroxidation and maintaining glutathione homeostasis [43,88,89,90]. Combined exercise and L-dopa therapy in Parkinson’s disease models demonstrates that exercise-induced NRF2 upregulation mitigates L-dopa-associated oxidative stress, illustrating the potential of non-pharmacological adjunctive approaches. Neurodegenerative disorders demonstrate responsiveness to non-pharmacological NRF2 activation, attributed to non-electrophilic mechanisms that upregulate glutathione peroxidase 4 (GPX4), glutathione reductase, HO-1, and manganese superoxide dismutase. Beyond physical exercise, electroacupuncture ameliorates motor impairment in models of hemiparkinsonism and Parkinson’s disease models through striatal NRF2 and HO-1 upregulation [88,90,91,92]. NRF2 activation effectively reduces symptomatology in models of vascular dementia using TMS, EA, and remote ischemic pre-conditioning [47,93,94], Huntington’s disease using TMS [95], and neuropsychiatric models including anxiety, depression, and PTSD using TMS, EA, and electroconvulsive therapy (ECT) [96,97,98,99]. Additionally, olive-oil-enriched diets and caloric restriction (CR) attenuate age-related degeneration through NRF2 upregulation and reduction of oxidative markers including malondialdehyde (MDA) and C-reactive protein (CRP), resulting in enhanced cognitive performance [100,101,102,103,104].

**Table 1:** Non-pharmacological NRF2 interventions investigated in physiological conditions.

Model	Intervention Type	Species	Intervention Parameters	Focal Organ/Cell	NRF2 Measurement Timing	NRF2 Modulation	Other Effects	Mechanism	Ref.
Healthy	Physical exercise	Mice	Treadmill running; 2 consecutive days; 60 min per day; 14 m/min; 10% slope	Heart	Directly after AES	AES: ↑ NRF2	NRF2−/− mice: ↓ GSH	ROS production	[63]
	Physical exercise	Mice	Treadmill running; 20 m/min; 5% slope; one or six hours	Hind limb	Directly after PE	6H PE: ↑ NRF2/ARE binding activity 1H PE: ↔ NRF2/ARE binding activity	6H: ↑ GCLm, GCLc, SOD1, SOD2, CAT, HO-1	ROS → KEAP1	[64]
	Physical exercise	Young human males	Cycling; 30 min at 70% VO2max OR 7 cycles at 90% VO2max	Peripheral blood mononuclear cell	Directly after PE	↑ NRF2 Intensity had no effect	Cycling for 7 cycles ↑ 8-isoprostanes and glutathione reductase in comparison to cycling for 30 min	Not specified	[65]
	Physical exercise	Mice	Muscle stimulation on the right leg, high intensity OR low intensity	Gastrocnemius and soleus muscles	30 min after muscle stimulation	High intensity: local and systemic ↑NRF2 Low intensity: systemic ↓ NRF2 local ↑ NRF2	↔ glutathione ↑ NQO1	KEAP1	[105]
	Physical exercise	Mice	Endurance exercise (EES): 90 min/per day for 2 days OR moderate exercise training (MET): 50 min/day for 6 weeks	Heart	Directly after PE	EES: ↑ NRF2 in young mice Prolonged MET: ↑ NRF2 in aging mice	MET: NQO1, HO-1, and GSR were similar in young and old mice	Not specified	[106]
	TMS	In vitro	One session of 10 series at 5, 10, and 15 Hz	THP-1-derived macrophages	4 h, 6 h, and 24 h	Time-dependent ↑ NRF2	5 min treatment: ↔ inflammatory factors Pre-treatment: ↓ IL-1β and TNF-β	KEAP1 p62	[44]
	TH	In vitro	8 h at 37 °C or 32 °C	HepG2-ARE stable cells	Directly after TH	↓ antioxidant genes after treatment with TH	Lower temperatures (27 °C and 22 °C): did not activate NRF2 and HIF1A pathways as efficiently as mild hypothermia	Possibly post-translational mechanism	[107]
	DR	Rats	Acute: single diving session Chronic: daily diving sessions for 4 weeks	Brain, kidney, lung	Directly after DR	↑ NRF2 phosphorylation and nuclear translocation	↑ GSH/GSSG ↑ SOD, ↑ HO-1, ↑ NQO1, ↓ 4HNE, ↓ nitrotyrosine, ↔ MDA	CGRP→ KEAP1/p62, AMPK, SIRT1, PI3K	[66]
	RIPreC	Rats	1 session of 60 min ischemia followed by 60 min reperfusion	Skin tissue	After flap surgery	↑ NRF2	Viable flap area was smaller in groups with serum transfer than those w/o RIC	Not specified	[108]
	PBM	In vitro	Bisphenol A (BPA) + photobiomodulation (PHT) wavelength: 660 + 10, output power: 35, energy density (J/cm^2^): 0.28, for 12 min	ADSCs	Directly after PBM	↑ NRF2	Low concentrations of BPA + PHT: induced autophagy	p62	[67]
	PBM	In vitro	100 mW, wavelength of 808 nm, spot area of 0.5 cm^2^, and irradiation at 1, 2, and 3 J/cm^2^s OR PBM + metformin	HPDLSCs	Directly after PBM	3 J/cm^2^ PBM + metformin: ↑ NRF2. Was more than 2 J/cm^2^ PBM + metformin	3 J/cm^2^ PBM: ↑ PIK3 3 J/cm^2^ PBM + metformin: ↓ TNF-α	KEAP1	[68]
	PBM	In vitro	Blue light-500 mW/cm^2^	A431 epidermoid carcinoma cells	Directly after PBM	↑ NRF2	Average volume of light-treated tumors was significantly lower than that in the untreated controls	Not specified	[109]
	PBM	In vitro	250 mW, 500 mW, 1000 mW for 30 s/8 h for 12 days	Schwann cells	Directly after PBM	↑ NRF2	250 mW and 500 Mw: ↓ apoptosis	PI3K/Akt signaling pathway	[110]
	Dietary change—CR	Rats	24 mo old with lifelong 40% CR	CMVECs	After establishment of CMVECs	↑ NRF2	↓ age-related impairment of angiogenic processes	miR-144	[103]
	Intermittent fasting	Mice	Mice fasted for 24 h	Skeletal gastrocnemius muscle	After fasting	↑ NRF2	↑ Cat, Gclc, Gclm, Gsr, HO-1 and Ucp3, GPX4	Not specified	[111]
	Intermittent fasting	Humans	Fasted during Ramadan. Daily fasting between 23–30 days	Blood sample	Directly before RIF and after Ramadan	↑ NRF2	↑ SOD2, TFAM ↓ SIRT3	ROS production	[69]
	Intermittent fasting	Mice, WT and Tlr4-/-	Food deprived for 24 h every other day, for 30 days	Hippocampus	After regimen	Tlr4-/-: ↓ NRF2 Tlr4-/- + IF: ↓ NRF2	Tlr4-/- + IF: ↓ memory, depressive-like behavior	Not specified	[71]
	Intermittent fasting	Mice with ZEB1 or ZEB2 knockout	Deprived of food but free access to water for 3 days			ZEB1-/- + IF: ↓ NRF2 ZEB12-/- + IF: ↑ NRF2		Not specified	[70]

**Table 2:** Non-pharmacological NRF2 interventions investigated in chronic pathological states.

Model	Intervention Type	Species	Intervention Parameters	Focal Organ/Cell	NRF2 Measurement Timing	NRF2 Modulation	Other Effects	Mechanism	Ref.
HFD-induced non-alcoholic fatty liver disease (NAFLD)	Physical exercise	Zebrafish	Placed in swimming tunnel. Exercised for 5 days per week	Liver	Fresh liver tissue samples	↑ NRF2	↓ oxidative stress and apoptosis	SIRT1/AMPK signaling	[43]
Hemiparkinsonism	Physical exercise pre-treatment	Mice	Treadmill, 6 weeks, 5 times/week, time-out on weekends	Striatum	Directly after hemiparkinsonism was induced	PE: ↑ NRF2	↓ nigrostriatal neurodegeneration, functional impairment, and supersensitivity of DA receptors	Not specified	[92]
Parkinsonism	Physical exercise pre-treatment	Rats	Treadmill, at 70% of maximal oxygen consumption for 60 min/day, 5 days/week for 4 weeks	Striatum, substantia nigra	Directly after parkinsonism was induced	↑ NRF2	↔ glutathione or ratio of GSSG	ROS production	[90]
	Physical exercise	Rats	After induction of PD. Treadmill, 30 min/day, 5 times a week for 4 weeks	Striatum	After behavioral tests	↑ NRF2	↑ NQO1 and TFAM	Not specified	[88]
A53T α-syn, related to PD	EA	A53T mice	4 weeks starting at 2 months of age at the 2t36 and SP6 acupoints. Intensity increased stepwise	Brain—midbrain and striatum		↑ NRF2	↑ HO-1 and glutamate-cysteine ligase modifier subunits	Not specified	[91]
Heart failure with preserved ejection fraction (HFpEF)	VNS (non-invasive)	Rats	20 Hz, 0.2 ms, 2 mA, daily for 30 min over a 4-week period	Subfornical organ, spinal trigeminal nucleus	After VNS	↑ NRF2	↑ NQO1	Not specified	[112]
Huntington’s disease	TMS	Rats	60 Hz, 0.7 mT, 2 h in the morning and 2 h in the afternoon, 8 consecutive days, starting 4 days before the first injection of 3-NP	Striatum	After ELFEF	↑ NRF2		Not specified	[95]
Vascular dementia	TMS	Rats	10 days, 10 Hz for 2 h per day	Brain tissue	After TMS	↑ NRF2	↑ GPx4 and learning memory ability	Not specified	[47]
	EA	Rats	Third day after 2VO. Administered at DU20 and ST36 acupoints for two weeks	Hippocampus	After EA	↑ NRF2	↓ microglia activation and cognitive deficits	Not specified	[94]
	RIPreC	Mice	Four 10-min cycles	Brain	After RIPreC	↑ NRF2	↑ glutathione reductase	Not specified	[93]
Senescence	TMS	SAMP89 mice	25 Hz for durations of 14 and 28 days	Hippocampus		↑ NRF2	↓ MDA, ↑ GPX4	Not specified	[46]
	Dietary change—olive oil phenolics	SAMP8 mice	Ate diet with high or low amounts of olive oil phenolics for 4.5 months	Heart		High olive oil phenolics: ↑ NRF2	High olive oil phenolics: ↑ GST, γ-GCS, NQO1, and PON2 mRNA l	SIRT1	[101]
	Dietary change—Med diet	Humans	4 weeks. Med diet, Med diet with CoQ (Med + CoQ), Western diet rich with saturated fats (SFA)	Blood samples—peripheral blood mononuclear cells	4 h after diet	Med + CoQ diet: ↑ cytoplasmic NRF2, ↓ nuclear NRF2 SFA diet: ↓ cytoplasmic NRF2, ↑ nuclear NRF2 Med diet: intermediate effects	↓ SOD1 and SOD2, TrxR, NADPH-oxidase (p22phox and p47phox subunits)	KEAP1	[102]
	Physical exercise	Rats	Treadmill exercise. 5 days/week for 6 weeks	Renal proximal tubules	48 h after PE	↑ NRF2	↓ MDA, CRP ↑ SOD1, IL-10	Not specified	[89]
CUMS	TMS	Rats	15 Hz for 15 min for 7 consecutive days	Hippocampus	After TMS	↑ NRF2	↓ depressive and anxiety-like behavior	Not specified	[96]
	EA pre-treatment	Rats	1 h before CUMS protocol. Administered at GV23 and GV16 acupoints, every other day for 4 weeks	Hippocampus	After inducing CUMS	↑NRF2	↓depressive behaviors, oxidative stress, and MDA	Not specified	[97]
	ECT	Rats	Once daily for 10 days at 100 Hz for 0.5 s at 80 mA	Hippocampus	After ECT	ECT: ↑ NRF2	ECT: ↓ depressive-like behaviors and hippocampal neuronal ferroptosis	BDNF	[98]
Enhanced single prolonged stress (ESPS)–PTSD	EA pre-treatment	Rats	Given at GV20 acupoint for 30 min daily (frequency: 2/15 Hz, intensity: 1 mA)	Hippocampus	14 days after ESPS	↑ NRF2	↑ HO-1, BDNF, AMPK, hippocampal neurogenesis ↓ anxiety-like behaviors	KEAP1	[99]
Sleep deprivation (SD)	TMS–cTBS	Mice	600 pulses of 3 stimuli of 40 s for 7 sessions	Hippocampus	After cTBS	↑ NRF2	↑ spatial learning and memory abilities ↓ oxidative stress, inflammation, and autophagy of hippocampal tissues	Not specified	[113]
Sepsis	EA	Rats	Given at ST36 0.5 mA and 15 Hz for 30 min once daily for five days	Hippocampus	After EA	↑ NRF2	EA: ↓ MDA	α7nAChR	[87]
	TH	Rats	10 h. Maintained at 32–33.9 °C	Lungs	5 days after model is induced	↑ NRF2	TH: ↑ GPX4 via the KEAP1/NRF2/SLC7A11 signaling pathway	PI3K/Akt/GSK3β signaling pathway	[114]
Diabetic encephalopathy	EA	Rats	30 min alternately at ST36 and EXB3 acupoints once a day for 4 weeks	Hippocampus	After EA	↑ NRF2	↑ HO-1 ↑ learning and memory abilities	Not specified	[84]
Complex regional pain syndrome type-I	EA	Rats	Given at ST36 and BL60 acupoints on a daily basis for 7 days	Hind paw	After EA	↑ NRF2	↓ mechanical pain response	Not specified	[85]
Ventilator-induced lung injury (VILI)	EA	Mice	Given at BL13 and ST36 acupoints five times a week for 2 weeks	Lung	After EA	↑ NRF2	↑ HO-1. ↓ activation of NLRP3 inflammasome	Not specified	[115]
Carcinogenesis	Dietary change—CR	Mice	CR mice were fed 40% less than control	Liver	After carcinogenesis	CR required NRF2 for protection against induced tumors	CR: ↑ HO-1, GCLC, GST A1, and GPx-1	Not specified	[104]
Obesity	Dietary change—diets with virgin olive oil (VOO)	Humans	Each group ingested one breakfast every 2 weeks, until completing the four breakfasts	Blood samples—peripheral blood mononuclear cells	After 12 h fasting and at 2 and 4 h after ingestion of each breakfast	4 h after VOO, SOX, or SOP: ↓ NRF2 4 h after SFO: ↑ NRF2	4 h after VOO: ↓ SOD1, GPx 4 h after SOX: ↓ GPx 4 h after SFO: ↑ NRF2 GSH ↑ higher with VOO, SOX, or SOP in comparison to SFO	Not specified	[116]
	Intermittent fasting	Humans	Ramadan fasting periods—28 to 30 consecutive days abstaining from food and drink from dawn to sunset	Blood samples	2–7 days before fasting and after Ramadan	IF: ↑ NRF2	IF: ↑ SOD2, TFAM, CD163	Not specified	[117]
Adriamycin (ADR)-induced nephropathy	Intermittent fasting	Rats	Alternate 24-h fasting and feeding periods for 8 weeks	Kidney	After regimen	↑ NRF2	↑ SIRT1, HO-1, ↑ AQP2	Not specified	[118]

The mechanistic basis for the largely beneficial non-pharmacological NRF2 activation in chronic cerebral dysfunction involves predominantly non-electrophilic pathways operating through both KEAP1-dependent and KEAP1-independent mechanisms. Physical exercise exhibits particularly diverse mechanistic profiles. In high-fat diet zebrafish models, swimming exercise upregulates SIRT1 expression and AMPK phosphorylation, subsequently enhancing p-AKT (protein kinase B) and NRF2 expression through non-electrophilic pathways involving NRF2 nuclear translocation and post-translational modifications [43], effectively attenuating hepatic lipid accumulation and steatosis. Conversely, in 1-methyl-4-phenylpyridine-induced parkinsonian rat models, striatal NRF2 activation occurs via exercise-induced mild oxidative stress, preserving nigrostriatal dopaminergic neurons [90]. This suggests concurrent electrophilic and non-electrophilic mechanisms, providing therapeutic benefit without electrophile-associated cytotoxicity. Electroacupuncture and Mediterranean dietary patterns demonstrate multi-mechanistic NRF2 activation in moderate cerebral dysfunction. Electroacupuncture activates NRF2 through α7nAChR in sepsis models [87] and via KEAP1 modulation in PTSD [91]. Mediterranean diets enhance KEAP1/NRF2 dissociation and SIRT1 activation in senescence models [101,102]. In contrast, electroconvulsive therapy and therapeutic hypothermia demonstrate singular mechanistic pathways in moderate cerebral dysfunction: electroconvulsive therapy modulates brain-derived neurotrophic factor (BDNF) in chronic unpredictable mild stress models [98,102], while TH activates the survival-mediated PI3K/Akt/GSK3β signaling cascade. Although additional interventions demonstrate efficacy in chronic disease contexts, their mechanistic characterization remains incomplete.

### 2.4. Non-Pharmacological NRF2 Activation in Acute Oxidative Stress Paradigms

Consistent with observations in chronic oxidative stress conditions, electrophilic activators demonstrate substantial translational limitations in acute, high-oxidative stress paradigms, contrasting markedly with the therapeutic benefits of non-pharmacological NRF2 activators. Sulforaphane administration in middle cerebral artery occlusion (MCAO) ischemia/reperfusion models produces a 1.3-fold increase in protein oxidation relative to vehicle controls [6]. Despite concomitant 2.5-fold HO-1 upregulation, lesion volume reduction reaches only 50% [6], compared to 70% reduction achieved with glutathione administration [7]. This phenomenon extends beyond sulforaphane; in stroke models, DMF fails to consistently reduce lesion volume or oxidative stress, potentially exacerbating lesion damage within three days of administration [13,14,15]. These findings indicate that electrophilic-activator-mediated NRF2 induction and subsequent antioxidant upregulation, including glutathione synthesis, yield limited therapeutic benefit under acute oxidative stress conditions. Furthermore, despite NRF2’s established role in oxidative stress and inflammatory modulation, DMF administration in traumatic brain injury models provides neither antioxidative nor anti-inflammatory benefits [16,17], paradoxically increasing cerebral lipid peroxidation [66]. Given the brain’s high lipid content, this effect poses particular neurotoxicological risks [25].

Conversely, non-pharmacological interventions demonstrate robust NRF2 activation efficacy in acute/traumatic pathologies characterized by rapid oxidative stress escalation, including ischemia/reperfusion (I/R) injury (cutaneous, hepatic, myocardial, pulmonary, retinal), cerebral ischemia, traumatic brain injury, intracerebral and subarachnoid hemorrhage, hemorrhagic shock, cardiac arrest/hypoxia, acute lung injury, and acute inflammation (Table 3, Figure 3). This therapeutic efficacy persists despite heightened stress sensitivity and increased probability of contraindications or diminished effectiveness in these contexts. Remote ischemic conditioning (RIC) represents a predominant intervention for I/R injury mitigation. Remote ischemic post-conditioning (RIPostC) activates NRF2 across cerebral, myocardial, renal, and retinal I/R models, while remote ischemic pre-conditioning (RIPreC) demonstrates age-dependent effects, upregulating NRF2 in young but not aged hearts following myocardial I/R injury [119,120,121,122,123,124]. Additional NRF2-mediated I/R protective interventions include: pre-ischemic exercise reducing stroke severity, therapeutic hypothermia attenuating cerebral cell death, electroacupuncture upregulating pulmonary HO-1 in pulmonary and cerebral I/R, olive oil dietary supplementation enhancing wound healing post cutaneous I/R, and combined vagus nerve stimulation with therapeutic hypothermia increasing hepatic antioxidant enzyme expression [125,126,127,128,129,130,131]. Following traumatic cardiac injury, secondary organ damage is ameliorated through NRF2 activation via RIPreC, therapeutic hypothermia (4–24 h), non-invasive vagus nerve stimulation, electroacupuncture pretreatment, and PBM [112,132,133,134,135,136,137,138]. Plasma-mediated effects of RIC, PBM, and dietary modifications demonstrate systemic NRF2 activation, reducing acute inflammatory responses in transfer experiments [139,140,141]. In traumatic brain injury, therapeutic hypothermia and median nerve stimulation enhance NRF2 expression, ameliorating cognitive deficits [142,143]. Intermittent fasting upregulates NRF2 following intracerebral hemorrhage and nephropathy [118,144]. Additionally, RIPreC attenuates neurological dysfunction in hemorrhagic shock through NRF2 activation [145], while acute pulmonary injury is modulated by electroacupuncture and therapeutic hypothermia-induced NRF2 expression [115,146].

**Table 3:** Non-pharmacological NRF2 interventions investigated in acute injury and traumatic pathologies.

Model	Intervention Type	Species	Intervention Parameters	Focal Organ/Cell	NRF2 Measurement Timing	NRF2 Modulation	Other Effects	Mechanism	Ref.
Cerebral ischemia—MCAO	Physical exercise	Rats	30-min treadmill training with either Constraint-induced movement therapy (CIMT) OR unconstrained exercise (UE)	Brain	Directly after PE	CIMT in comparison to UE: ↑ NRF2	CIMT in comparison to UE: ↓ MDA	KEAP1	[147]
	Physical exercise—pre-conditional	Mice	Housed for 6 weeks in a cage with a running wheel	Brain—microglia	Directly after PE	PE: ↑ NRF2	PE w/o stroke: ↔ CB2R, P2Y12, mafk, and p-NRF2 PE w/stroke: ↑ CB2R, P2Y12, mafk, and p-NRF2	CB2R	[125]
	EA	Rats	30 min for 7 days; continuous 2/100 Hz, ~2–4 V and 0.5~1.5 mA at the PC6, DU26, SP6, and DU20 acupoints	Brain—cerebral cortex	Directly after EA	↑ NRF2	↑GPX4 and SLC7A11, ↓neuronal damage and neuronal mitochondrial injury, ↓ iron	Not specified	[128]
	EA pre-treatment	Mouse	30 min, 1 mA, 2/15 Hz at the GV20 acupoint	Brain—cerebral cortex	2 h after MCAO	↑ NRF2	↑ HO-1 and NQO1	GSK-3β	[42]
	TH	Rats	Cold condition (4 °C), isolated cortical temperature of 33 ± 1 °C during ischemia	Brain	24 h after reperfusion	↑ NRF2	↓ neurological deficit and cerebral cell death	PPARs	[126]
	RIPreC	Rats	LRIC (3 cycles) was applied every day up to 14 days before MCAO	Brain	24 h after reperfusion	↑ NRF2	↑ SOD1 and HO-1	Not specified	[148]
	RIPostC	Mice	4 cycles lasting 40 min in total and continued every 12 h until execution (treated twice daily for 1, 3, or 7 days)	Brain—cortex	After behavioral testing	↑ NRF2	↑ HO-1, NLRP3, cleaved caspase-1, TAC, SOD, GSH/GSSG levels, neurological function, ↓ MDA	KEAP1	[119]
	RIPostC	Mice	3 cycles immediately after stroke onset	Brain—cerebral cortex	24 h after reperfusion	↑ NRF2	↑ in neurological outcome, HO-1, NQO-1, SOD, ↓ MDA	Not specified	[120]
Subarachnoid hemorrhage	Physical exercise	Rats	Pre-conditioning exercise, treadmill, 30 min/day, 5 days/week for 3 weeks	Brain—motor cortex		↑ NRF2	↓ neurological deficits, sensorimotor dysfunction, and consciousness disorder	Not specified	[149]
Intracerebral hemorrhage (ICH)	Intermittent fasting	Mice	Every-other-day feeding	Microglia of ipsilateral basal ganglia	Days 1, 2	Day 1 and 3 after ICH: ↑ NRF2 Day 7: returned to baseline	↓ CD16+Iba-1+ microglia activation, IL-1β, TNF-α	SIRT3	[144]
I/R on skin	Dietary change = soybean oil and olive oil	Mice	6 weeks subjected to either regimen	Blood samples—proteins of wound lysate	14 days after second IR cycle	↑ NRF2	Promoted wound closure at 7, 10, and 14 days	Not specified	[129]
MIRI	Delayed RIPreC (DRIPC)	Rats	4 cycles once per day for 3 days before heart isolation	Heart—left ventricle	30 min after stabilized perfusion	↑ NRF2	↑ HO-1	Did not activate PKB/Akt or ERK 1/2	[150]
	RIPreC	Rats	Four cycles for a total of 40 min	Heart	24 h after RIPreC	↑ NRF2 in young hearts, ↓ in old hearts		HIF-1 α	[151]
	VNS	Rats	Intensity: 0.5 V, frequency: 2.5 Hz, pulse width: 5 ms, duration: 5 min	Heart	After I/R	↑ NRF2	↑ GRPR	KEAP1	[152]
	RIPostC	Mice	3 cycles at the start of reperfusion period	Heart	After 2 h if cardiac reperfusion	↑ NRF2	↑ Akt, HO-1, SOD1, ↓ MDA	STAT3	[121]
Hepatic I/R	TH	Rats	Induced by the superfusion of cooled saline at 26 °C onto the ischemic lobes	Liver	Directly after TH	↓ NRF2	↑ NQO1	KEAP1	[153]
	VNS	Rats	20 Hz for 0.1 millisecond	Liver	Directly after VNS	↑ NRF2	↑ HO-1	Not specified	[130]
	VNS	Rats	Interval of 1 s, a duration of 1 ms, and a frequency of 5 Hz	Lung	After 6 h of reperfusion	↑ NRF2	↓ MPO and MDA	Not specified	[131]
	VNS	Rats	20 Hz, 0.2 ms in duration	Kidneys	After I/R	↑ NRF2	↑ HO-1	Not specified	[154]
Lung I/R	EA	Rabbits	15 min once a day for 5 days at BL13 and ST36 acupoints	Lung	After I/R	↑ NRF2	↑ SOD, GPx, and CAT ↓ MDA	p38 MAPK	[127]
Renal I/R	RIPostC	Mice	3 cycles of 5 min during the reperfusion period	Kidney	24 h after reperfusion	↑ NRF2	↑ HO-1, SOD ↓ MDA	TOPK/Akt	[122]
	RIPostC	Rats	Began on second post-operative day, lasted 10 min	Retina	After RIPostC	↑ NRF2	↑ HO-1, ↓ GFAP	Not specified	[123]
Hemorrhagic shock/resuscitation (S/R)	RIPreC	Mice, zebrafish	Mice underwent 4 cycles for 10 min. Zebrafish were treated with RIC blood	Liver of mice. Plasma from zebrafish	After hemorrhage (S/R)	RIPreC for mice and zebrafish: ↑ NRF2		KEAP1 and ERK 1/2	[145]
Tailfin-cut inflammation	RIPostC	Zebrafish	Fish were treated with RIC plasma injection from wildtype mice (4 cycles of 5 min) and NRF2-knockout mice			↑ NRF2	↑ hmox1a, ↓ neutrophil migration	ROS	[139]
Endotoxic-shock-induced acute lung injury	EA	Rabbits	Performed throughout the operating steps for 6 h during the experimental day at ST36 and BL13 acupoints	Lung	After EA	↑ NRF2	↑ HO-1, SOD, GPx, and CAT, ↓ MDA	Not specified	[146]
Hydrogen-peroxide-induced oxidative stress	RIPreC	In vitro	Human umbilical vein endothelial cells (HUVECs) were treated with rat sera. Rats underwent 3 cycles of 10 min	HUVECs	After sera was injected	↑ NRF2	↓ MDA	Not specified	[155]
Inflammation—stimulation with 2,4-dinitrochlorobenzene (DNCB)	PBM	In vitro	660 nm (red light) or 520 nm (green light), 20 min after DNCB treatment. Exposure time of 250 s	Primary human KCs	After PBM	↑ NRF2	↑ HO-1, NQO1, and GCLC	Not specified	[141]
DSS-induced acute colitis	EA	Mice	30 min at ST36 acupoints	Colon—macrophages	After EA	↑ NRF2	EA: ↑ NRF2 HO-1, ↓ NLRP3/IL-1β activation	Not specified	[86]
TBI	MNS	Rats	300 microseconds at 40 Hz for 20 s/min. Continued 8 h per day for 2 weeks	Hippocampus	After MNS	↑ NRF2	↑ GPX4, SLC7A11, VEGF	Not specified	[142]
	TH	Mice	4 h at 37 degrees Celsius	Cortex	24 h after TBI	↑ NRF2	↑ HO-1 and NQO-1 ↓ MDA	Not specified	[143]
Hypothermic circulatory arrest (HCA)	RIPreC	Piglets	4 cycles of 5-min ischemia followed by 5-min reperfusion	Cortex, hippocampus, thalamus, brainstem, cerebellum	After RIPreC	↑ NRF2	↓ 8-OHdG	HIF-1-α	[133]
Cardiac hypoxia	PBM		8 W/cm^2^–12 W/cm^2^ 0.15–250 s, varied energy intensity	H9C2 cardiomyocytes	After PBM	↑ NRF2	↓ SOD2, PGC-1α	p62	[134]
Cardiac arrest	TH	Rats	4 h after return of spontaneous circulation	Hippocampus	After TH	↑ NRF2	↓ MDA, caspase-3 ↑ SOD	GSK-3β	[135]
	TH	Minipigs	24 h after ROSC. TH was maintained for 12 h	Frontal cortex	After TH	↑ NRF2	↓ MDA	Oxidative stress	[136]
	TH	Rats	Hypothermia was maintained at 33 ± 0.5 °C for four hours	Lumber spinal cord—motor neurons and glial-like cells	24 h after CA	↑ NRF2	↑ HO-1	Not specified	[137]
CA-induced renal I/R	TH	Rats	Administered for 2, 4, 6 h	Kidney	After TH	↑ NRF2, more ↑ with time	↑ HO-1, ↓ MDA	Not specified	[138]
Cardiopulmonary bypass	EA pre-treatment	Rats	EA at PC6 and LI4 acupoints for 30 min before CPB	Lung	After EA	↑ NRF2	↓ pulmonary neutrophil infiltrations ↓ TNF-α, IL-18, and IL-1β	ROS	[132]

Limited mechanistic investigations of non-pharmacological NRF2 activation in acute contexts reveal a multi-mechanistic regulatory paradigm. RIPostC demonstrates pathway-specific activation across different pathologies: in myocardial I/R injury, RIPostC induces STAT3 phosphorylation, subsequently activating Akt and upregulating NRF2 expression [121]. Conversely, in renal I/R models, T-LAK-cell-originated protein kinase (TOPK) mediates NRF2 expression, preventing renal dysfunction and tubular necrosis [122]. TOPK phosphorylation inactivates phosphatase and tensin homolog (PTEN), thereby activating PI3K/Akt signaling and NRF2 regulation. In cerebral ischemia, RIPostC downregulates KEAP1, attenuating neuronal damage [119]. RIPreC employs combined mechanisms, inducing ROS and electrophile production for KEAP1 dissociation while modulating HIF-1α in aged myocardium [124], integrating multiple non-electrophilic pathways. Temporal analysis reveals mechanistic complexity: delayed RIPreC effects (24–72 h post-administration) occur independently of PKB/Akt and ERK1/2 pathways, despite documented ERK1/2 dependence in other studies [145,150]. Furthermore, NRF2 activation by sera from RIC-treated rats proceeds independently of MEK/ERK and PI3K/Akt pathways [155], suggesting context-dependent activation mechanisms requiring further investigation. Electroacupuncture integrates multiple non-electrophilic pathways, including GSK-3β downregulation and p38 MAPK upregulation [42,127], concurrent with electrophilic NRF2 activation. This dual mechanism likely explains clinical efficacy without organ toxicity despite electrophilic components [126,135,136,137,138]. Other non-pharmacological interventions demonstrate singular mechanistic pathways in acute/traumatic conditions: intermittent fasting upregulates sirtuin 3 (SIRT3), exercise modulates cannabinoid receptor 2 (CB2R), and PBM increases PGC-1α [125,134,140,144]. Therapeutic hypothermia employs dual mechanisms, modulating PPARs and PI3K/Akt/GSK3β signaling, exemplifying the multi-mechanistic potential of non-pharmacological activators.

## 3. Discussion and Future Perspectives

### 3.1. Multi-Mechanistic NRF2 Activation: A Strategy to Circumvent Constraints of Current Electrophilic and Non-Electrophilic Activators

Multi-mechanistic NRF2 activation strategies demonstrate superior therapeutic potential compared to singular pathway approaches. Classical electrophilic activators employ exclusively electrophilic mechanisms [1,2,3,4], whereas interventions including physical exercise, remote ischemic conditioning, intermittent fasting, and electroacupuncture integrate ROS generation with protein–protein interaction inhibition, KEAP1 degradation, protein stability modulation, or post-translational modifications (Figure 3), thereby reducing potential adverse events. Physical exercise exemplifies this multi-mechanistic approach, combining electrophilic ROS production with SIRT1/AMPK signaling modulation [43], where non-electrophilic pathways potentially counterbalance electrophilic toxicity. Therapeutic hypothermia similarly integrates electrophilic ROS generation with PPAR targeting and post-translational modifications [107,126,153].

Non-pharmacological activators inherently employ multiple NRF2-targeting mechanisms, potentially yielding more nuanced or pronounced effects than current pharmacological agents (Figure 3). This suggests that combining interventions with distinct mechanistic profiles could address limitations inherent to both classical electrophilic and non-pharmacological activators. Despite potential cytotoxicity, electrophilic activation remains among the most potent NRF2 activation methods, providing a greater enhancement to NRF2 bioavailability [37,38]. However, electrophilic mechanisms constrain pharmacological intervention utility. Subtherapeutic pharmacological dosing to enhance NRF2 bioavailability [37,38], combined with non-pharmacological activators to improve NRF2 efficiency, presents a viable strategy. This approach shows particular promise when employing interventions like electroacupuncture or dietary restriction, which utilize multiple non-electrophilic pathways, including MAPK/p38, α7nAChR, p62, AMPK, and SIRT1 [87,127].

Non-pharmacological activator combinations may address limitations of both classical electrophilic activators and individual non-pharmacological interventions. Dietary modifications alone produce insufficient sustained NRF2 activation [102], with miR-144 and miR-146a silencing showing limited efficacy [102,156]. However, combination with pharmacological coenzyme Q10 produces synergistic effects exceeding either intervention alone. Similarly, combining dietary interventions with PBM, which targets NRF2 availability through PGC-1α rather than efficiency [134], may yield enhanced NRF2 activation. Multi-factorial approaches thus exemplify the adjunctive potential of non-pharmacological NRF2 activators, combining interventions with complementary mechanisms to overcome individual limitations and optimize therapeutic efficacy. Realizing this potential requires characterizing individual intervention limitations and examining interintervention interactions. Despite these challenges, the therapeutic promise for NRF2 activation and cerebral dysfunction treatment justifies intensive investigation of these multi-mechanistic strategies.

### 3.2. Cell-Specific Oxidative Stress Vulnerability and NRF2 Utilization: Mechanistic Advantages of Non-Electrophilic Activation Pathways

**Figure 4:**
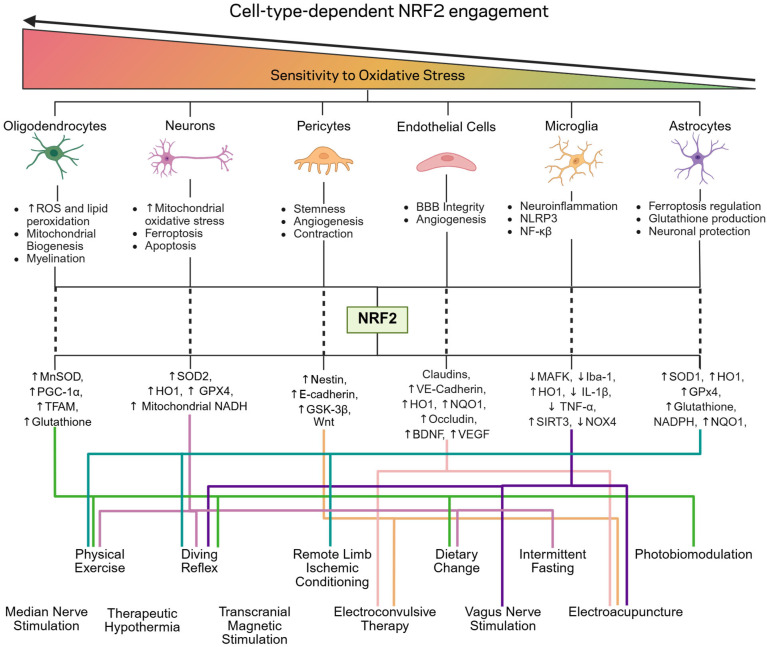
Cell-type-dependent NRF2 engagement. Neurons, oligodendrocytes, astrocytes, microglia, and vascular cells possess distinct NRF2 regulatory baselines that reflect their intrinsic oxidative stress susceptibility. Consequently, the optimal route of NRF2 induction varies across cell types and, by extension, across neurological diseases dominated by those cells. Although direct cell-specific data for non-pharmacological interventions are not yet available, the signaling pathways they recruit provide a rationale for therapeutic matching. Modalities such as physical exercise and DR activation, which primarily engage neuronal and oligodendroglial NRF2 signaling, may be preferable for disorders characterized by parenchymal injury. In contrast, interventions that robustly influence vascular NRF2 networks, e.g., electroacupuncture or electroconvulsive stimulation, may prove more advantageous in pathologies driven by cerebrovascular dysfunction. (Created with BioRender.com).

NRF2 activator efficacy in the brain exhibits cell-type-dependent variability determined by differential cellular interactions. Distinct brain cell populations demonstrate heterogeneous oxidative stress responses and NRF2 pathway utilization, reflecting cell-specific oxidative stress sensitivities (Figure 4) and variable basal intracellular NRF2 concentrations [50]. Therefore, the therapeutic efficacy of NRF2-targeted interventions in cerebral pathology is determined not by absolute NRF2 activation levels but by cell-type-specific responses. This phenomenon becomes particularly relevant under chronic administration or acute/traumatic conditions, where electrophilic compound limitations manifest. Cell-type-specific characterization of non-pharmacological interventions remains limited. Nevertheless, mechanistic profiles and documented downstream antioxidative effects enable prediction of cell-type-specific therapeutic suitability. This analysis systematically examines cerebral cellular populations and their NRF2 utilization patterns, stratified by oxidative stress sensitivity, correlating specific activation mechanisms and antioxidative outcomes with appropriate cellular targets. This framework facilitates identification of pathological contexts where non-pharmacological NRF2 activators demonstrate optimal therapeutic potential, particularly through predominant non-electrophilic pathway activation.

#### 3.2.1. Oligodendrocytes

Oligodendrocytes function as the primary myelinating cells of the central nervous system, essential for myelination, remyelination processes, and metabolic efficiency [157]. Among cerebral cell populations, oligodendrocyte precursor cells and mature oligodendrocytes demonstrate maximal oxidative stress susceptibility, exceeding that of cortical neurons and astrocytes [158] (Figure 4). This heightened vulnerability results from elevated ROS generation, increased metabolic demands, and enhanced lipid peroxidation susceptibility [157]. Consequently, oligodendrocytes exhibit pronounced NRF2 dependence, maintaining the highest basal NRF2 expression among cerebral cell types [159]. NRF2-mediated glutathione synthesis prevents metabolic cell death [160], ameliorating mitochondrial dysfunction and endoplasmic reticulum stress [161], while promoting mitochondrial biogenesis through PGC-1α activation [162]. Therefore, glutathione depletion by electrophilic activators [9] poses particular risks to oligodendrocyte viability.

The critical role of PGC-1α specifically implicates PBM, dietary restriction, dietary modifications, and physical exercise as therapeutic interventions. PBM directly upregulates PGC-1α [134], while the latter three modulate AMPK and/or SIRT1, essential regulators of PGC-1α biosynthesis [163]. These interventions, particularly those exhibiting dual AMPK/SIRT1 modulation, demonstrate enhanced potential for mediating oligodendrocyte oxidative stress resistance. This suggests therapeutic applicability in neurodegenerative pathologies including multiple sclerosis, Alzheimer’s disease, and Parkinson’s disease, where oxidative-stress-induced oligodendrocyte precursor cell differentiation failure impairs remyelination and adaptive myelination [164]. The chronic nature of these pathologies particularly favors non-electrophilic non-pharmacological NRF2 activators over electrophilic compounds, given the documented drawbacks of electrophilic activators in chronic administration [74,76].

#### 3.2.2. Neurons

Neuronal death in cerebral dysfunction occurs predominantly through oxidative-stress-mediated excitotoxicity, apoptosis, and ferroptosis [50]. Neurons exhibit the second-highest oxidative stress vulnerability among brain cells (Figure 4). Neurons demonstrate exceptional mitochondrial dependence for bioenergetic requirements, exceeding other cell types. Consequently, oxidative-stress-induced mitochondrial damage significantly contributes to neuronal death, suggesting heightened vulnerability to electrophilic activators that disrupt mitochondrial bioenergetics [1,2,3,4,5,6,7,20,21,22,23,24,26,27,28,29,30]. Oxidative stress induces mitochondrial membrane hyperpermeabilization [26,27,28,29], initiating caspase-dependent apoptosis through ROS release, resulting in DNA fragmentation and cellular death [30]. Lipid peroxidation products, particularly 4-hydroxynonenal [22,23,24], generated through glutathione peroxidase inhibition and glutathione depletion [20,21], exacerbate this process—representing key mechanisms of electrophilic activator toxicity, including sulforaphane [165].

Therapeutic targeting of mitochondrial oxidative stress and lipid peroxidation represents optimal neuroprotective strategy. This requires selective upregulation of SOD2 for mitochondrial ROS scavenging and HO-1 and GPX4 for ferroptosis inhibition [166]. Additionally, PGC-1α modulation provides therapeutic benefit through mitochondrial biogenesis regulation [163]. Among interventions enhancing PGC-1α synthesis, dietary restriction and physical exercise, which concurrently modulate HO-1, glutathione, and/or SOD2, demonstrate superior neuroprotective potential [64,66]. These approaches show particular relevance in neurodegenerative pathologies including Alzheimer’s and Parkinson’s diseases, characterized by oxidative-stress-mediated neuronal dysfunction [60,167,168]. Physical exercise’s efficacy in Parkinson’s disease exemplifies this principle, where multi-mechanistic NRF2 activation neutralizes potential electrophilic activation-associated toxicity [88,90,92].

#### 3.2.3. Pericytes and Endothelial Cells

Pericytes and endothelial cells constitute critical components of cerebral microvasculature, BBB integrity, and angiogenic processes [169] (Figure 4). Pericytes demonstrate temporal-dependent oxidative stress sensitivity: acute exposure induces relaxation, 30-min exposure triggers constriction, and prolonged exposure results in cellular death [170]. Endothelial cells similarly exhibit heightened oxidative stress vulnerability, manifesting as disrupted intracellular signaling, compromised nitric oxide synthesis, and increased vascular permeability [171,172,173,174]. NRF2 directly mediates endothelial integrity and BBB protection, preventing post-cerebral-insult leukocyte infiltration through HO-1-dependent mechanisms that inhibit endothelial apoptosis [27,173]. In pericyte–endothelial interactions, NRF2 promotes angiogenesis via BDNF upregulation [28], an effect replicated by electroacupuncture [99] and electroconvulsive therapy [98,102]. Additionally, NRF2 regulates pericyte stemness through nestin and E-cadherin mRNA upregulation, facilitating pericyte differentiation [175].

Paradoxically, DMF increases leukocyte infiltration in experimental traumatic brain injury, suggesting BBB dysfunction and endothelial/pericyte damage despite NRF2 upregulation [16]. Sulforaphane demonstrates dose-dependent inhibition of endothelial and pericyte proliferation, suppressing tube formation [176] and disrupting intercellular communication through angiogenic factor inhibition, indicating NRF2 pathway interference. Conversely, the NRF2–E-cadherin–pericyte differentiation axis suggests electroacupuncture therapeutic potential, given its documented NRF2 activation [99] and E-cadherin/GSK-3β upregulation [177]. Oxidative-stress-induced pericyte contraction dysregulates cerebral blood flow, exacerbating hypoperfusion [178]. The Wnt signaling involvement particularly implicates electroacupuncture efficacy, given its pathway-mediated effects [177,179]. Electroacupuncture’s mechanistic profile, integrating Wnt/β-catenin, E-cadherin, and GSK-3β modulation [99,177,179], suggests specific efficacy in vascular degenerative pathologies including vascular cognitive impairment and dementia, characterized by microvascular loss requiring regenerative intervention [180]. Similarly, dietary restriction’s beneficial effects in vascular dementia models, including pericyte preservation, angiogenesis, and vascular maintenance [180], may result from potent non-electrophilic NRF2 activation [66].

#### 3.2.4. Microglia

Microglia function as the brain’s resident immune cells, mediating tissue repair, neurogenesis, soluble factor release, synaptic remodeling, and phagocytosis [181]. Microglial cells maintain higher NRF2 expression than neurons, exhibiting enhanced ARE activity and elevated NRF2 transcript levels, with functionality predominantly oriented toward inflammatory modulation [181,182,183]. In microglia, NRF2 inhibits NFκB signaling, modulating innate and adaptive immune responses while suppressing proinflammatory cytokine and chemokine release (Figure 4). This mechanism prevents NLRP3 inflammasome assembly, disrupting inflammatory cascades and inhibiting pyroptotic cell death [184]. NRF2-mediated NADPH oxidase 4 (NOX4) blockade specifically prevents NLRP3 formation [185,186,187,188]. This pathway particularly implicates Mediterranean dietary patterns in microglial NRF2 modulation; elevated polyphenol intake downregulates NOX via NRF2, attenuating oxidative stress and preventing inflammatory accumulation [102]. Furthermore, downstream effectors HO-1 and NQO1 specifically reduce NLRP3 formation and caspase-1 expression, suppressing inflammatory cytokine release [189]. These mechanisms suggest that interventions including electroacupuncture [42], vagus nerve stimulation [112], and dietary restriction [66] represent appropriate therapeutic strategies for neuroinflammatory conditions including multiple sclerosis, encephalitis, meningitis, and acute trauma-induced inflammation [190,191,192].

#### 3.2.5. Astrocytes 

Astrocytes represent the predominant glial cell population within the brain, executing diverse supportive functions including nutrient transport, BBB maintenance, ionic homeostasis, and tissue repair processes [193] (Figure 4). Under controlled culture conditions, astrocytes demonstrate enhanced NRF2 expression and superior oxidative stress resilience, mediating protective responses against oxidative insults [194]. During oxidative stress exposure, astrocytes upregulate antioxidant molecules including HO-1 and glutathione, facilitating protective neuronal–astrocytic intercellular communication [50]. Astrocytic NRF2 overexpression potentially maintains neuronal metabolic homeostasis following mitochondrial complex II inhibition and glutamatergic excitation, mediating HO-1 upregulation while suppressing ferroptosis through SOD1 and GPX4 enhancement. Therefore, astrocyte-targeted therapeutic strategies may confer neuroprotection through paracrine mechanisms. Interventions selectively upregulating HO-1 and SOD1 demonstrate preferential astrocytic targeting, including physical exercise [64] and remote ischemic conditioning [148]. Astrocyte-focused therapeutic approaches demonstrate particular relevance in acute traumatic pathologies, where neuronal oxidative stress generation resulting from bioenergetic dysfunction represents a critical pathophysiological mechanism [195].

### 3.3. Therapeutic Applications of Non-Pharmacological NRF2 Activators in Neurodegenerative Pathologies and Psychological Disorders

Current therapeutic strategies for neurodegenerative diseases remain predominantly symptomatic, highlighting the critical need for disease-modifying interventions. Oxidative stress represents a fundamental pathogenic mechanism across neurodegenerative disorders [60,167,168]. In Parkinson’s disease, α-synuclein oligomer aggregation induces cytotoxicity, ferroptosis, and mitochondrial ROS generation, resulting in dopaminergic neuronal depletion [196,197,198,199]. In amyotrophic lateral sclerosis, by contrast, excessive ROS generation, arising from compromised antioxidant defenses and pathogenic mutations, serves as a principal mechanism driving motor neuron degeneration [200,201]. Similarly, in Alzheimer’s disease pathogenesis, β-amyloid peptide aggregates interact with mitochondria, compromising mitochondrial function and enhancing ROS production [202,203,204], initiating progressive neuronal death cascades [205,206]. Tau accumulation, in tauopathies, leads to similar structural and metabolic neuronal and microenvironmental compromise, by disrupting physiological microtubule function [207,208,209,210], all the way to mitochondrial impairment. 

Comparable oxidative stress mechanisms characterize Huntington’s disease and multiple sclerosis pathophysiology, suggesting conserved pathogenic pathways across neurodegenerative conditions [168]. The pivotal role of ROS implicates dysregulated NRF2 signaling in disease progression [167], indicating that NRF2 upregulation and subsequent antioxidant pathway enhancement may confer neuroprotection. However, clinical application of pharmacological NRF2 activators remains constrained, necessitating alternative therapeutic approaches. Non-pharmacological NRF2 activators targeting oxidative stress mechanisms may provide preventive benefits not yet comprehensively characterized. These interventions demonstrate sustained efficacy under chronic administration without the organ toxicity and mitochondrial dysfunction associated with pharmacological agents, supporting their application as long-term prophylactic strategies. Pre-clinical evidence substantiates the potential therapeutic efficacy, in neurodegenerative conditions, for several interventions, including TMS [47], electroacupuncture [94], remote ischemic pre-conditioning [93], and DR [180] in vascular dementia; TMS in Huntington’s disease [95]; and physical exercise in Parkinson’s disease [88,90,92].

Non-pharmacological NRF2 activators also demonstrate therapeutic potential extending beyond neurodegenerative pathologies to encompass psychological disorders. NRF2 functions as an upstream regulator of critical factors implicated in psychological dysfunction pathogenesis [211], including oxidative stress, neurotransmitter systems targeted by conventional psychotropic medications, apoptosis, and neuroinflammation. This multi-factorial modulation positions NRF2 as a promising therapeutic target for psychological disorders [177], as demonstrated by pharmacological interventions including CDDO-Im in post-stroke depression models [177] and TP-500 in intracerebral hemorrhage models [212]. Correspondingly, TMS and electroconvulsive therapy demonstrate preliminary efficacy in attenuating depressive and anxiety-like behaviors in chronic unpredictable mild stress models through hippocampal NRF2 upregulation [96,98]. Electroacupuncture pre-treatment in enhanced single prolonged stress models of post-traumatic stress disorder, modulating AMPK, BDNF, and KEAP1, upregulates NRF2 activation and HO-1 expression, correlating with reduced anxiety manifestations [99]. These findings suggest that additional non-pharmacological interventions enhancing cerebral NRF2 expression may demonstrate efficacy in psychological disorders, including dietary restriction (increasing brain-wide NRF2 activation, particularly hippocampal) [66], vagus nerve stimulation (enhancing NRF2 and NAD(P)H:quinone oxidoreductase expression in the subfornical organ and spinal trigeminal nucleus) [112], and remote ischemic pre-conditioning [93]. Given psychological dysfunction prevalence affecting 5–15% of the global population and 30–50% of individuals with cerebral pathology [211], novel interventions targeting fundamental pathophysiological mechanisms warrant prioritized investigation, particularly considering their demonstrated potential in acute cerebral injury contexts.

## 4. Conclusions

This review addresses two central topics: (i) the paradox that classical electrophilic NRF2 activators can be less effective than individual downstream antioxidants—and may, under certain conditions, exacerbate pathology—and (ii) the prospect that non-pharmacological interventions provide a viable solution. A systematic evaluation of the literature reveals that non-canonical, non-electrophilic pathways circumvent the shortcomings of electrophilic agents, particularly their limited neuroprotective efficacy, especially when combined in a multi-mechanistic manner. We delineate the multi-mechanistic activation profiles characteristic of non-pharmacological modalities, identifying this mechanistic plurality as critical to their therapeutic capacity. The combination of non-electrophilic action and pathway multiplicity affords cell-type-specific regulation, enabling precision strategies across a broad spectrum of neurological disorders. Furthermore, the convergence of multiple activation mechanisms lays the groundwork for combinatorial therapeutic approaches that surpass the constraints of traditional electrophilic compounds and single-mechanism non-pharmacological tactics. Collectively, this evidence underscores the promise of non-electrophilic, multi-mechanistic, non-pharmacological NRF2 activators and outlines essential research directions to advance their clinical translation.

## Data Availability

No new data was generated.

## Abbreviations

The following abbreviations are used in this manuscript:

ADSC	Adipose-tissue-derived stem cell
α7nACHr	Alpha7 nicotinic acetylcholine receptor
AD	Alzheimer’s disease
AMPK	AMP-activated protein kinase
ARE	Antioxidant response element
BDNF	Brain-derived neurotrophic factor
CR	Caloric restriction
CB2R	Cannabinoid receptor 2
CA	Cardiac arrest
CMVECs	Cerebromicrovascular endothelial cells
CUMS	Chronic unpredictable mild stress
cTBS	Continuous theta burst stimulation
CRP	C-reactive protein
DMF	Dimethyl fumarate
DR	Diving reflex
EA	Electropuncture
ECT	Electroconvulsive therapy
ERK	Extracellular signal-regulated kinase
GPX4	Glutathione peroxidase 4
GSK3β	Glycogen synthase kinase 3 beta
HO-1	Heme oxygenase-1
HFD	High-fat diet
I/R	Ischemia/reperfusion
KEAP1	Kelch-like ECH-associated protein 1
MDA	Malondialdehyde
MNS	Median nerve stimulation
MCAO	Middle cerebral artery occlusion
MIRI	Myocardial ischemia–reperfusion injury
NQO1	NAD(P)H:quinone oxidoreductase 1
NRF2	Nuclear factor erythroid 2-related factor 2
PD	Parkinson’s disease
PI3K	Phosphoinositide-3-kinase
PBM	Photobiomodulation
PTSD	Post-traumatic stress disorder
Akt	Protein kinase B
p38 MAPK	p38 mitogen-activated protein kinase
ROS	Reactive oxygen species
RIC	Remote ischemic conditioning
RIPreC	Remote ischemic pre-conditioning
RIPostC	Remote ischemic post-conditioning
STAT3	Signal transducer and activator of transcription 3
SIRT1	Sirtuin 1
SIRT3	Sirtuin 3
SOD1	Superoxide dismutase 1
SOD2	Superoxide dismutase 2
TH	Therapeutic hypothermia
TOPK	T-LAK cell-originated protein kinase
TMS	Transcranial magnetic stimulation
TBI	Traumatic brain injury
VNS	Vagus nerve stimulation
